# 2-[(Cyclo­hex-3-en-1-ylmeth­oxy)meth­yl]-6-phenyl-1,2,4-triazine-3,5(2*H*,4*H*)-dione

**DOI:** 10.1107/S1600536812021198

**Published:** 2012-05-19

**Authors:** Nasser R. El-Brollosy, Mohamed I. Attia, Ali A. El-Emam, Seik Weng Ng, Edward R. T. Tiekink

**Affiliations:** a Department of Pharmaceutical Chemistry, College of Pharmacy, King Saud University, Riyadh 11451, Saudi Arabia; b Department of Chemistry, Faculty of Science, Tanta University, Tanta 31527, Egypt; c Department of Chemistry, University of Malaya, 50603 Kuala Lumpur, Malaysia; d Chemistry Department, Faculty of Science, King Abdulaziz University, PO Box 80203 Jeddah, Saudi Arabia

## Abstract

In the title 1,2,4-triazine derivative, C_17_H_19_N_3_O_3_, the heterocyclic ring is planar (r.m.s. deviation = 0.040 Å) and effectively coplanar with the adjacent phenyl ring [dihedral angle = 4.5 (2)°] but almost perpendicular to the (cyclo­hex-3-en-1-ylmeth­oxy)methyl residue [N—N—C—O torsion angle = 71.6 (5)°], so that the mol­ecule has an ‘L’ shape. Supra­molecular chains along [001] are formed in the crystal *via* N—H⋯O hydrogen bonds where the acceptor O atom is the ether O atom. The adjacent carbonyl O atom forms a complementary C—H⋯O contact resulting in the formation of a seven-membered {⋯HNCO⋯HCO} heterosynthon; the second carbonyl O atom forms an intra­molecular C—H⋯O contact. Chains are connected into a supra­molecular layer in the *ac* plane by π–π inter­actions [ring centroid–centroid distance = 3.488 (3) Å]. The central atom in the –CH_2_CH_2_C(H)= residue of the cyclo­hexene ring is disordered over two sites, with the major component having a site-occupancy factor of 0.51 (2).

## Related literature

For the potential medicinal applications of 1,2,4-triazines, see: Ban *et al.* (2010 ▶); Irannejad *et al.* (2010 ▶); Sangshetti & Shinde (2010 ▶). For the synthesis, see: El-Brollosy (2008 ▶).
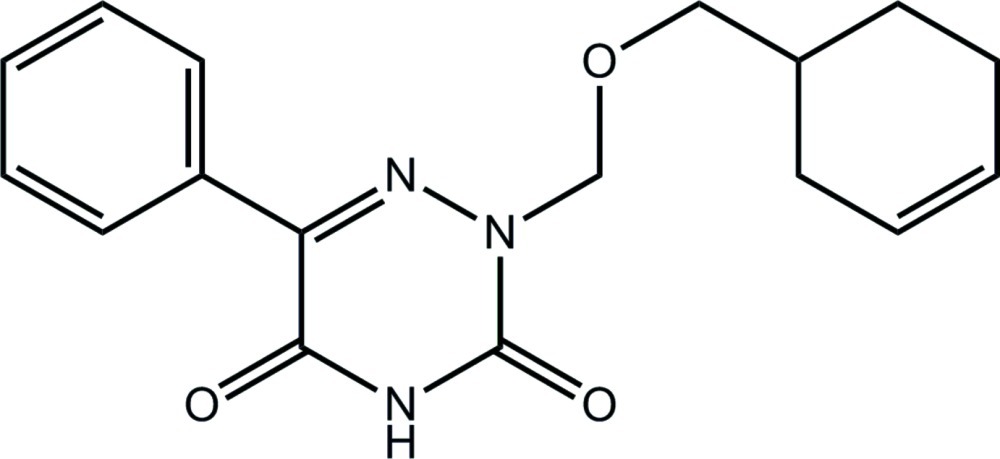


## Experimental

### 

#### Crystal data

C_17_H_19_N_3_O_3_
*M* *_r_* = 313.35
Monoclinic, 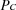

*a* = 4.7924 (6) Å
*b* = 13.7083 (19) Å
*c* = 11.8293 (15) Å
β = 101.538 (12)°
*V* = 761.43 (17) Å^3^
*Z* = 2
Mo *K*α radiation
μ = 0.10 mm^−1^
*T* = 100 K
0.35 × 0.15 × 0.03 mm

#### Data collection

Agilent SuperNova Dual diffractometer with an Atlas detector
Absorption correction: multi-scan (*CrysAlis PRO*; Agilent, 2011 ▶) *T*_min_ = 0.806, *T*_max_ = 1.000
4230 measured reflections
1757 independent reflections
1158 reflections with *I* > 2σ(*I*)
*R*_int_ = 0.078

#### Refinement

*R*[*F* ^2^ > 2σ(*F* ^2^)] = 0.061
*wR*(*F* ^2^) = 0.133
*S* = 1.04
1757 reflections
212 parameters
4 restraints
H-atom parameters constrained
Δρ_max_ = 0.24 e Å^−3^
Δρ_min_ = −0.30 e Å^−3^

### 

Data collection: *CrysAlis PRO* (Agilent, 2011 ▶); cell refinement: *CrysAlis PRO*; data reduction: *CrysAlis PRO*; program(s) used to solve structure: *SHELXS97* (Sheldrick, 2008 ▶); program(s) used to refine structure: *SHELXL97* (Sheldrick, 2008 ▶); molecular graphics: *ORTEP-3* (Farrugia, 1997 ▶) and *DIAMOND* (Brandenburg, 2006 ▶); software used to prepare material for publication: *publCIF* (Westrip, 2010 ▶).

## Supplementary Material

Crystal structure: contains datablock(s) global, I. DOI: 10.1107/S1600536812021198/su2425sup1.cif

Structure factors: contains datablock(s) I. DOI: 10.1107/S1600536812021198/su2425Isup2.hkl

Supplementary material file. DOI: 10.1107/S1600536812021198/su2425Isup3.cml

Additional supplementary materials:  crystallographic information; 3D view; checkCIF report

## Figures and Tables

**Table 1:** Hydrogen-bond geometry (Å, °)

*D*—H⋯*A*	*D*—H	H⋯*A*	*D*⋯*A*	*D*—H⋯*A*
N1—H1⋯O3^i^	0.88	2.00	2.877 (5)	174
C2—H2⋯O1	0.95	2.21	2.880 (7)	127
C10—H10*B*⋯O2^ii^	0.99	2.46	3.352 (6)	150

Symmetry codes: (i) 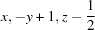; (ii) 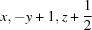.

**Fig. 1.**
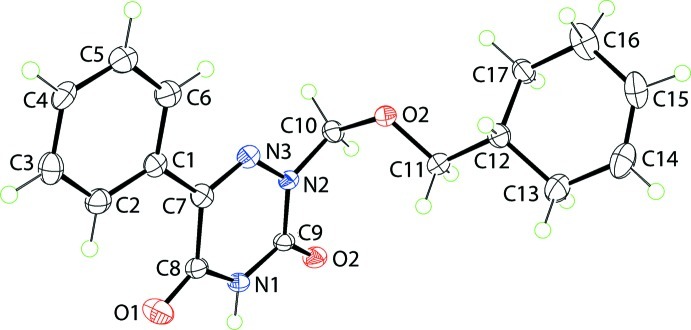
The molecular structure of (I) showing the atom-labelling scheme and displacement ellipsoids at the 50% probability level. Only the major component of the disordered C16 atom is shown.

**Fig. 2.**
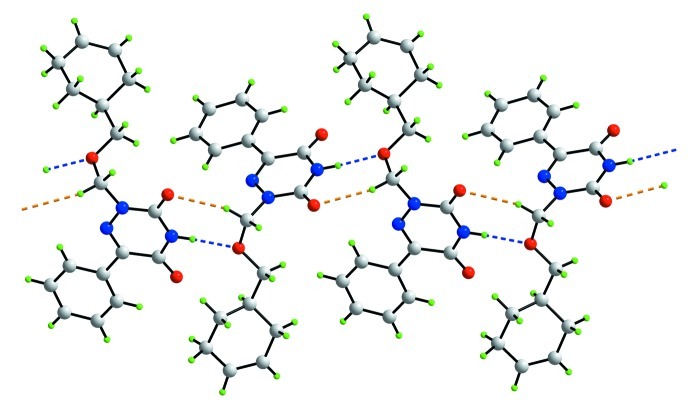
A view of the supramolecular chain in (I) mediated by N—H···O hydrogen bonding and reinforced by C—H···O interactions, shown as blue and orange dashed lines, respectively.

**Fig. 3.**
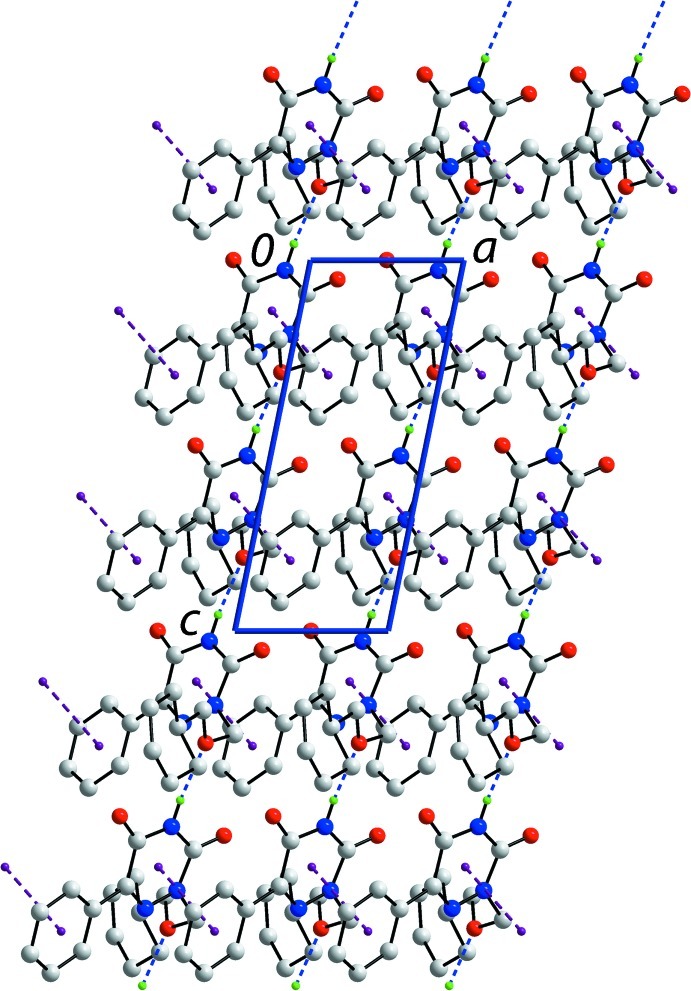
A view of the supramolecular layer parallel to the ac plane in (I). The N—H···O and π—π interactions are shown as blue and purple dashed lines, respectively. Hydrogen atoms not participating in intermolecular interactions have been omitted.

**Fig. 4.**
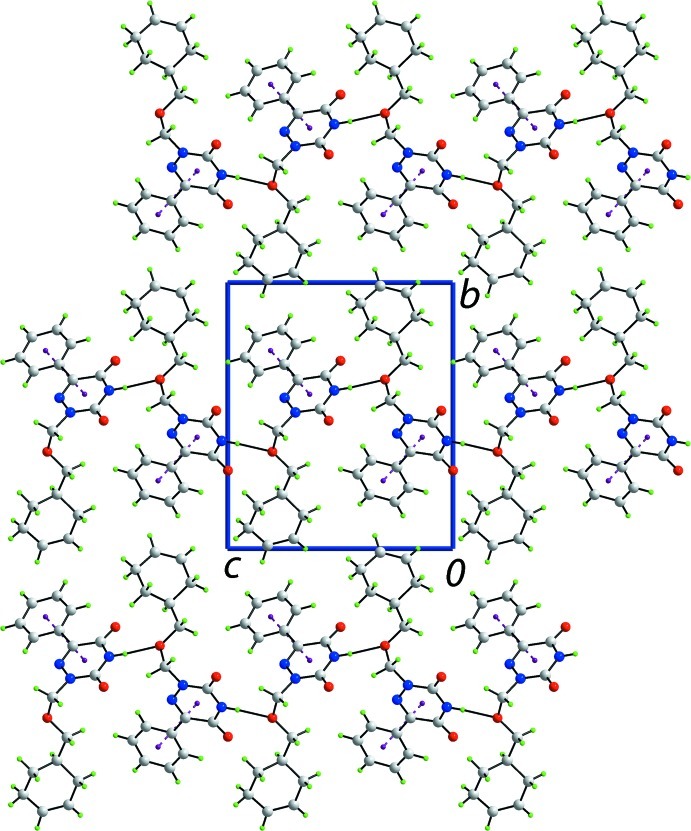
A view in projection down the a axis of the unit-cell contents for (I). The N—H···O and π—π interactions are shown as blue and purple dashed lines, respectively.

## supplementary crystallographic information

### Comment

Several 1,2,4-triazines have been shown to exhibit herbicidal, anti-viral, anti-microbial, anti-inflammatory, anti-malarial, anti-cancer, anti-proliferative and neuroprotective activities (Ban *et al.*, 2010; Irannejad *et al.*, 2010; Sangshetti & Shinde, 2010). The title compound (I), was originally synthesized as a potential anti-microbial agent (El-Brollosy, 2008). Herein, we describe the results of its crystal structure determination.

The six ring atoms comprising the 2,4-dihydro-1,2,4-triazine-3,5-dione residue in (I), Fig. 1, are co-planar with a r.m.s. = 0.040 Å; the maximum deviations from their least-squares plane being 0.035 (5) Å for the C7 atom and -0.039 (5) Å for the C8 atom. The dihedral angle between this ring and the attached phenyl ring of 4.5 (2)° is consistent with an almost co-planar relationship. The (cyclohex-3-en-1-ylmethoxy)methyl residue lies perpendicular to the rest of the molecule as seen in the value of the N3—N2—C10—O3 torsion angle of 71.6 (5)°, so that to a first approximation the molecule has an *L*-shape.

The crystal structure features supramolecular chains along [001]. These are mediated by, perhaps surprisingly, N—H···O hydrogen bonds where the O atom is the ether O atom, rather than carbonyl O atoms. The adjacent carbonyl-O2 atom forms a complementary C—H···O contact resulting in the formation of a seven-membered {···HNCO···HCO} heterosynthon, Fig. 2 and Table 1. The carbonyl-O1 atom forms an intramolecular C—H..O interaction. The chains are connected into supramolecular layers in the *ac* plane by π—π interactions [ring centroid(N1–N3,C7–C9)···centroid(C1–C6)^i^ = 3.488 (3) Å and tilt angle = 4.5 (2)°; symmetry code: (i) x+1, y, z], Fig. 3. Supramolecular layers stack along the *b* axis with no specific interactions between them, Fig. 4.

### Experimental

5-Phenyl-6-azauracil (0.189 g, 1 mmol) was stirred in dry acetonitrile (15 ml) under nitrogen and *N*,*O*-bis-trimethylsilylacetamide (0.87 ml, 3.5 mmol) was added. After a clear solution was obtained (10 min), the mixture was cooled to 223 K and trimethylsilyl trifluoromethanesulphonate (0.18 ml, 1 mmol) was added followed by the drop wise addition of bis(3-cyclohexen-1-ylmethoxy)methane (0.472 g, 2 mmol). The reaction mixture was stirred at room temperature for 5 h. The reaction was quenched by addition of sat. *aq*. NaHCO_3_ solution (5 ml). The mixture was evaporated under reduced pressure and the residue was extracted with ether (3 *x* 50 ml). The combined ether fractions were collected, dried (MgSO_4_) and evaporated under reduced pressure. The residue was purified on a silica gel column using 1:5 petroleum ether / chloroform to give the title compound in 64% (0.199 g) yield. Colourless crystals were obtained upon crystallization from its ethanol solution (El-Brollosy, 2008).

### Refinement

H-atoms were placed in calculated positions [N—H = 0.88 and C—H = 0.95 to 0.99 Å, *U*_iso_(H) = 1.2*U*_eq_(N,*C*)] and were included in the refinement in the riding model approximation. The amino H-atom was refined freely. In the absence of significant anomalous scattering effects, 799 Friedel pairs were averaged in the final refinement. The C16 atom of the cyclohexene ring was disordered over two positions. From anisotropic refinement, the major component of the disorder had a site occupancy factor = 0.51 (2). The pairs of the respective C15—C16/C16'—C17 bond lengths were restrained to be within 0.01 Å of each other; the anisotropic displacement parameters for the disordered atoms were constrained to be equal.

### Crystal data

**Table:**

C_17_H_19_N_3_O_3_	*F*(000) = 332
*M**_r_* = 313.35	*D*_x_ = 1.367 Mg m^−^^3^
Monoclinic, *P**c*	Mo *K*α radiation, λ = 0.71073 Å
Hall symbol: P -2yc	Cell parameters from 801 reflections
*a* = 4.7924 (6) Å	θ = 2.3–27.5°
*b* = 13.7083 (19) Å	µ = 0.10 mm^−^^1^
*c* = 11.8293 (15) Å	*T* = 100 K
β = 101.538 (12)°	Plate, colourless
*V* = 761.43 (17) Å^3^	0.35 × 0.15 × 0.03 mm
*Z* = 2	

### Data collection

**Table:**

Agilent SuperNova Dual diffractometer with an Atlas detector	1757 independent reflections
Radiation source: SuperNova (Mo) X-ray Source	1158 reflections with *I* > 2σ(*I*)
Mirror monochromator	*R*_int_ = 0.078
Detector resolution: 10.4041 pixels mm^-1^	θ_max_ = 27.5°, θ_min_ = 2.3°
ω scan	*h* = −5→6
Absorption correction: multi-scan (*CrysAlis PRO*; Agilent, 2011)	*k* = −17→17
*T*_min_ = 0.806, *T*_max_ = 1.000	*l* = −15→15
4230 measured reflections	

### Refinement

**Table:**

Refinement on *F*^2^	Primary atom site location: structure-invariant direct methods
Least-squares matrix: full	Secondary atom site location: difference Fourier map
*R*[*F*^2^ > 2σ(*F*^2^)] = 0.061	Hydrogen site location: inferred from neighbouring sites
*wR*(*F*^2^) = 0.133	H-atom parameters constrained
*S* = 1.04	*w* = 1/[σ^2^(*F*_o_^2^) + (0.0421*P*)^2^] where *P* = (*F*_o_^2^ + 2*F*_c_^2^)/3
1757 reflections	(Δ/σ)_max_ = 0.002
212 parameters	Δρ_max_ = 0.24 e Å^−^^3^
4 restraints	Δρ_min_ = −0.30 e Å^−^^3^

### Special details

**Table:**

Geometry. All e.s.d.'s (except the e.s.d. in the dihedral angle between two l.s. planes) are estimated using the full covariance matrix. The cell e.s.d.'s are taken into account individually in the estimation of e.s.d.'s in distances, angles and torsion angles; correlations between e.s.d.'s in cell parameters are only used when they are defined by crystal symmetry. An approximate (isotropic) treatment of cell e.s.d.'s is used for estimating e.s.d.'s involving l.s. planes.
Refinement. Refinement of *F*^2^ against ALL reflections. The weighted *R*-factor *wR* and goodness of fit *S* are based on *F*^2^, conventional *R*-factors *R* are based on *F*, with *F* set to zero for negative *F*^2^. The threshold expression of *F*^2^ > σ(*F*^2^) is used only for calculating *R*-factors(gt) *etc*. and is not relevant to the choice of reflections for refinement. *R*-factors based on *F*^2^ are statistically about twice as large as those based on *F*, and *R*- factors based on ALL data will be even larger.

### Fractional atomic coordinates and isotropic or equivalent isotropic displacement parameters (Å^2^)

**Table:**

	*x*	*y*	*z*	*U*_iso_*/*U*_eq_	Occ. (<1)
O1	0.4970 (7)	0.7062 (3)	0.4998 (3)	0.0321 (9)	
O2	1.2011 (7)	0.4812 (3)	0.5519 (3)	0.0245 (8)	
O3	0.9583 (7)	0.3645 (2)	0.8023 (3)	0.0228 (8)	
N1	0.8476 (9)	0.5948 (3)	0.5278 (4)	0.0205 (9)	
H1	0.8696	0.6052	0.4567	0.025*	
N2	0.9682 (7)	0.5123 (3)	0.7000 (3)	0.0190 (10)	
N3	0.7856 (7)	0.5669 (3)	0.7480 (3)	0.0194 (9)	
C1	0.4436 (9)	0.6924 (4)	0.7489 (4)	0.0201 (11)	
C2	0.2789 (10)	0.7697 (4)	0.6974 (5)	0.0253 (12)	
H2	0.2844	0.7872	0.6202	0.030*	
C3	0.1041 (10)	0.8224 (4)	0.7579 (5)	0.0275 (13)	
H3	−0.0072	0.8754	0.7221	0.033*	
C4	0.0957 (11)	0.7960 (4)	0.8709 (5)	0.0244 (12)	
H4	−0.0211	0.8315	0.9125	0.029*	
C5	0.2553 (10)	0.7192 (4)	0.9223 (5)	0.0260 (12)	
H5	0.2458	0.7011	0.9989	0.031*	
C6	0.4310 (11)	0.6673 (4)	0.8634 (5)	0.0251 (12)	
H6	0.5428	0.6148	0.9004	0.030*	
C7	0.6321 (9)	0.6348 (4)	0.6886 (4)	0.0205 (11)	
C8	0.6423 (10)	0.6503 (4)	0.5652 (4)	0.0209 (11)	
C9	1.0207 (10)	0.5252 (4)	0.5896 (4)	0.0185 (11)	
C10	1.1326 (10)	0.4421 (4)	0.7787 (4)	0.0217 (11)	
H10A	1.2888	0.4159	0.7441	0.026*	
H10B	1.2188	0.4752	0.8517	0.026*	
C11	0.8986 (11)	0.2912 (4)	0.7141 (5)	0.0252 (12)	
H11A	1.0778	0.2701	0.6920	0.030*	
H11B	0.7715	0.3182	0.6449	0.030*	
C12	0.7570 (10)	0.2050 (4)	0.7590 (4)	0.0221 (11)	
H12	0.5764	0.2283	0.7801	0.027*	
C13	0.6801 (13)	0.1266 (4)	0.6644 (5)	0.0376 (14)	
H13A	0.5451	0.1545	0.5980	0.045*	
H13B	0.8544	0.1070	0.6373	0.045*	
C14	0.5488 (12)	0.0383 (4)	0.7073 (6)	0.0365 (14)	
H14	0.4158	−0.0003	0.6557	0.044*	
C15	0.6234 (12)	0.0133 (4)	0.8234 (6)	0.0360 (14)	
H15	0.5694	−0.0490	0.8468	0.043*	
C16	0.779 (5)	0.0777 (12)	0.9093 (9)	0.032 (5)	0.51 (2)
H16A	0.9165	0.0382	0.9644	0.039*	0.51 (2)
H16B	0.6429	0.1066	0.9529	0.039*	0.51 (2)
C16'	0.837 (5)	0.0637 (12)	0.9049 (10)	0.032 (5)	0.49
H16C	1.0033	0.0199	0.9266	0.039*	0.49 (2)
H16D	0.7602	0.0758	0.9753	0.039*	0.49 (2)
C17	0.9405 (10)	0.1602 (4)	0.8654 (5)	0.0253 (12)	
H17A	1.1188	0.1347	0.8463	0.030*	
H17B	0.9912	0.2105	0.9261	0.030*	

### Atomic displacement parameters (Å^2^)

**Table:**

	*U*^11^	*U*^22^	*U*^33^	*U*^12^	*U*^13^	*U*^23^
O1	0.032 (2)	0.039 (2)	0.026 (2)	0.0098 (18)	0.0082 (17)	0.0077 (19)
O2	0.0274 (18)	0.027 (2)	0.0197 (19)	0.0025 (16)	0.0073 (16)	−0.0009 (16)
O3	0.032 (2)	0.0200 (19)	0.0175 (18)	−0.0002 (16)	0.0074 (16)	−0.0005 (15)
N1	0.028 (2)	0.021 (2)	0.014 (2)	0.0002 (18)	0.0081 (18)	0.0006 (17)
N2	0.024 (2)	0.018 (2)	0.015 (2)	0.0027 (18)	0.0042 (19)	−0.0017 (19)
N3	0.016 (2)	0.022 (2)	0.021 (2)	−0.0021 (18)	0.0038 (18)	−0.0020 (19)
C1	0.017 (2)	0.020 (3)	0.023 (3)	−0.006 (2)	0.004 (2)	−0.002 (2)
C2	0.029 (3)	0.024 (3)	0.023 (3)	−0.007 (2)	0.005 (2)	−0.002 (2)
C3	0.022 (3)	0.023 (3)	0.037 (3)	0.002 (2)	0.005 (3)	−0.002 (3)
C4	0.027 (3)	0.022 (3)	0.026 (3)	−0.003 (2)	0.011 (2)	−0.008 (2)
C5	0.025 (3)	0.029 (3)	0.025 (3)	−0.006 (2)	0.008 (2)	−0.002 (2)
C6	0.024 (3)	0.026 (3)	0.026 (3)	−0.005 (2)	0.006 (2)	−0.001 (3)
C7	0.017 (3)	0.021 (3)	0.023 (3)	−0.004 (2)	0.004 (2)	0.001 (2)
C8	0.025 (3)	0.021 (3)	0.017 (3)	−0.002 (2)	0.005 (2)	−0.001 (2)
C9	0.022 (3)	0.020 (3)	0.014 (3)	−0.005 (2)	0.004 (2)	0.000 (2)
C10	0.024 (3)	0.021 (3)	0.021 (3)	−0.005 (2)	0.005 (2)	0.002 (2)
C11	0.035 (3)	0.022 (3)	0.019 (2)	−0.001 (2)	0.005 (2)	−0.003 (2)
C12	0.021 (2)	0.021 (3)	0.024 (3)	0.003 (2)	0.004 (2)	0.001 (2)
C13	0.047 (3)	0.027 (3)	0.033 (3)	−0.004 (3)	−0.006 (3)	−0.004 (3)
C14	0.034 (3)	0.030 (3)	0.048 (4)	−0.011 (3)	0.012 (3)	−0.011 (3)
C15	0.033 (3)	0.021 (3)	0.053 (4)	−0.003 (3)	0.007 (3)	0.004 (3)
C16	0.014 (8)	0.029 (6)	0.055 (5)	0.013 (6)	0.008 (4)	0.012 (4)
C16'	0.014 (8)	0.029 (6)	0.055 (5)	0.013 (6)	0.008 (4)	0.012 (4)
C17	0.028 (3)	0.022 (3)	0.025 (3)	−0.002 (2)	0.002 (2)	0.005 (2)

### Geometric parameters (Å, º)

**Table:**

O1—C8	1.207 (6)	C10—H10A	0.9900
O2—C9	1.210 (5)	C10—H10B	0.9900
O3—C10	1.415 (6)	C11—C12	1.510 (7)
O3—C11	1.435 (6)	C11—H11A	0.9900
N1—C9	1.375 (6)	C11—H11B	0.9900
N1—C8	1.385 (6)	C12—C17	1.514 (7)
N1—H1	0.8800	C12—C13	1.543 (8)
N2—N3	1.359 (5)	C12—H12	1.0000
N2—C9	1.390 (5)	C13—C14	1.499 (8)
N2—C10	1.456 (6)	C13—H13A	0.9900
N3—C7	1.301 (6)	C13—H13B	0.9900
C1—C2	1.387 (7)	C14—C15	1.390 (9)
C1—C6	1.410 (7)	C14—H14	0.9500
C1—C7	1.486 (6)	C15—C16'	1.435 (11)
C2—C3	1.406 (7)	C15—C16	1.437 (11)
C2—H2	0.9500	C15—H15	0.9500
C3—C4	1.392 (7)	C16—C17	1.521 (9)
C3—H3	0.9500	C16—H16A	0.9900
C4—C5	1.371 (7)	C16—H16B	0.9900
C4—H4	0.9500	C16'—C17	1.519 (10)
C5—C6	1.391 (7)	C16'—H16C	0.9900
C5—H5	0.9500	C16'—H16D	0.9900
C6—H6	0.9500	C17—H17A	0.9900
C7—C8	1.486 (6)	C17—H17B	0.9900
			
C10—O3—C11	115.1 (3)	O3—C11—H11B	109.8
C9—N1—C8	127.1 (4)	C12—C11—H11B	109.8
C9—N1—H1	116.4	H11A—C11—H11B	108.3
C8—N1—H1	116.4	C11—C12—C17	112.5 (4)
N3—N2—C9	125.1 (4)	C11—C12—C13	110.4 (4)
N3—N2—C10	114.1 (4)	C17—C12—C13	109.5 (4)
C9—N2—C10	120.6 (4)	C11—C12—H12	108.1
C7—N3—N2	120.7 (4)	C17—C12—H12	108.1
C2—C1—C6	118.6 (5)	C13—C12—H12	108.1
C2—C1—C7	122.8 (4)	C14—C13—C12	111.9 (5)
C6—C1—C7	118.6 (4)	C14—C13—H13A	109.2
C1—C2—C3	120.9 (5)	C12—C13—H13A	109.2
C1—C2—H2	119.6	C14—C13—H13B	109.2
C3—C2—H2	119.6	C12—C13—H13B	109.2
C4—C3—C2	119.3 (5)	H13A—C13—H13B	107.9
C4—C3—H3	120.3	C15—C14—C13	119.4 (5)
C2—C3—H3	120.3	C15—C14—H14	120.3
C5—C4—C3	120.3 (5)	C13—C14—H14	120.3
C5—C4—H4	119.9	C14—C15—C16'	123.6 (6)
C3—C4—H4	119.9	C14—C15—C16	122.8 (6)
C4—C5—C6	120.7 (5)	C14—C15—H15	118.6
C4—C5—H5	119.6	C16'—C15—H15	115.8
C6—C5—H5	119.6	C16—C15—H15	118.6
C5—C6—C1	120.2 (5)	C15—C16—C17	116.2 (8)
C5—C6—H6	119.9	C15—C16—H16A	108.2
C1—C6—H6	119.9	C17—C16—H16A	108.2
N3—C7—C1	117.0 (4)	C15—C16—H16B	108.2
N3—C7—C8	120.4 (4)	C17—C16—H16B	108.2
C1—C7—C8	122.6 (4)	H16A—C16—H16B	107.4
O1—C8—N1	120.0 (4)	C15—C16'—C17	116.5 (8)
O1—C8—C7	126.4 (5)	C15—C16'—H16C	108.2
N1—C8—C7	113.6 (4)	C17—C16'—H16C	108.2
O2—C9—N1	123.3 (4)	C15—C16'—H16D	108.2
O2—C9—N2	124.0 (4)	C17—C16'—H16D	108.2
N1—C9—N2	112.6 (4)	H16C—C16'—H16D	107.3
O3—C10—N2	111.0 (4)	C16'—C17—C12	116.0 (7)
O3—C10—H10A	109.4	C16—C17—C12	109.6 (8)
N2—C10—H10A	109.4	C16'—C17—H17A	96.5
O3—C10—H10B	109.4	C16—C17—H17A	109.8
N2—C10—H10B	109.4	C12—C17—H17A	109.7
H10A—C10—H10B	108.0	C16'—C17—H17B	115.5
O3—C11—C12	109.3 (4)	C16—C17—H17B	109.7
O3—C11—H11A	109.8	C12—C17—H17B	109.7
C12—C11—H11A	109.8	H17A—C17—H17B	108.2
			
C9—N2—N3—C7	3.3 (6)	N3—N2—C9—N1	−5.5 (6)
C10—N2—N3—C7	177.7 (4)	C10—N2—C9—N1	−179.5 (4)
C6—C1—C2—C3	0.4 (7)	C11—O3—C10—N2	79.3 (5)
C7—C1—C2—C3	−179.8 (5)	N3—N2—C10—O3	71.6 (5)
C1—C2—C3—C4	−0.4 (7)	C9—N2—C10—O3	−113.8 (4)
C2—C3—C4—C5	−0.3 (8)	C10—O3—C11—C12	170.0 (4)
C3—C4—C5—C6	1.0 (8)	O3—C11—C12—C17	−59.4 (5)
C4—C5—C6—C1	−1.0 (7)	O3—C11—C12—C13	177.9 (4)
C2—C1—C6—C5	0.3 (7)	C11—C12—C13—C14	177.9 (5)
C7—C1—C6—C5	−179.5 (4)	C17—C12—C13—C14	53.4 (6)
N2—N3—C7—C1	−178.5 (4)	C12—C13—C14—C15	−29.4 (7)
N2—N3—C7—C8	3.3 (7)	C13—C14—C15—C16'	−4.5 (16)
C2—C1—C7—N3	177.0 (5)	C13—C14—C15—C16	12.3 (16)
C6—C1—C7—N3	−3.2 (6)	C14—C15—C16—C17	−19 (3)
C2—C1—C7—C8	−4.9 (7)	C16'—C15—C16—C17	79 (3)
C6—C1—C7—C8	174.9 (4)	C14—C15—C16'—C17	12 (3)
C9—N1—C8—O1	−177.0 (5)	C16—C15—C16'—C17	−79 (3)
C9—N1—C8—C7	4.6 (7)	C15—C16'—C17—C16	79 (3)
N3—C7—C8—O1	174.8 (5)	C15—C16'—C17—C12	15 (2)
C1—C7—C8—O1	−3.2 (8)	C15—C16—C17—C16'	−78 (3)
N3—C7—C8—N1	−6.9 (6)	C15—C16—C17—C12	43 (2)
C1—C7—C8—N1	175.1 (4)	C11—C12—C17—C16'	−170.4 (12)
C8—N1—C9—O2	−178.0 (5)	C13—C12—C17—C16'	−47.1 (12)
C8—N1—C9—N2	1.1 (7)	C11—C12—C17—C16	177.0 (10)
N3—N2—C9—O2	173.6 (4)	C13—C12—C17—C16	−59.8 (11)
C10—N2—C9—O2	−0.4 (7)		

### Hydrogen-bond geometry (Å, º)

**Table:**

*D*—H···*A*	*D*—H	H···*A*	*D*···*A*	*D*—H···*A*
N1—H1···O3^i^	0.88	2.00	2.877 (5)	174
C2—H2···O1	0.95	2.21	2.880 (7)	127
C10—H10*B*···O2^ii^	0.99	2.46	3.352 (6)	150

Symmetry codes: (i) *x*, −*y*+1, *z*−1/2; (ii) *x*, −*y*+1, *z*+1/2.
